# High-Speed Interrogation for Large-Scale Fiber Bragg Grating Sensing

**DOI:** 10.3390/s18020665

**Published:** 2018-02-24

**Authors:** Chenyuan Hu, Wei Bai

**Affiliations:** 1 MOE Key Laboratory of Fundamental Physical Quantities Measurement & Hubei Key Laboratory of Gravitation and Quantum Physics, School of Physics, Huazhong University of Science and Technology, Wuhan 430074, China; chenyuanhu@hust.edu.cn; 2 School of Information Engineering, Hubei University of Chinese Medicine, Wuhan 430065, China

**Keywords:** fiber Bragg gratings, fiber optics sensors, modulation, multiplexing

## Abstract

A high-speed interrogation scheme for large-scale fiber Bragg grating (FBG) sensing arrays is presented. This technique employs parallel computing and pipeline control to modulate incident light and demodulate the reflected sensing signal. One Electro-optic modulator (EOM) and one semiconductor optical amplifier (SOA) were used to generate a phase delay to filter reflected spectrum form multiple candidate FBGs with the same optical path difference (OPD). Experimental results showed that the fastest interrogation delay time for the proposed method was only about 27.2 us for a single FBG interrogation, and the system scanning period was only limited by the optical transmission delay in the sensing fiber owing to the multiple simultaneous central wavelength calculations. Furthermore, the proposed FPGA-based technique had a verified FBG wavelength demodulation stability of ±1 pm without average processing.

## 1. Introduction

Large-scale fiber Bragg grating (FBG) sensing arrays have attracted considerable interest because of their electromagnetic immunity, low crosstalk and strong multiplexing capacity [1,2,3]. The phase-mask online writing technique used to write weak FBGs from the draw tower eliminates large losses, low mechanical strength, etc. in the process of large-scale FBG networking (usually 0.1 dB loss with one welding joint). What’s more, the low reflectivity (<−30 dB) of weak FBGs effectively suppresses the accumulative noise, including the spectrum effect of shadow and the crosstalk between sensing cells [4,5]. However, as the capacity of sensing cells becomees larger and larger, the speed of FBG interrogation, especially with respect to gaining access to any given sensing cell along the fiber array, has become the main factor affecting the performance and application of FBG sensing systems [6,7]. Y. M. Wang et al. experimentally demonstrated a serial time division multiplexing (TDM) sensor network with more than 500 weak FBGs. As the position of FBG sensors in this system was located by ultra-high-speed sampling to reconstruct the reflection spectrum of each FBG throughout the entire scanning period in the time domain, it took a relatively longer time—generally over 10 s—to detect the result of a single FBG sensor [8]. Y. B. Dai et al. theoretically implemented a wavelength and time division multiplexing array with over 1000 sensors on one single fiber [9]. However, the speed of the optical spectrum analyzer (OSA) module and the personal computer are difficult to improve. K. Cui et al. calculated the phase in a field-programmable gate array (FPGA) in real time for a time division multiplexing sensor array [10]. However, both light source and sensor signal demodulation were present at the both ends of the sensing system. Ai et al. improved the spatial resolution to centimeters in a twin-grating FBG hybrid TDM and frequency-division multiplexing (FDM) sensing network [11]. However, the demodulation speed and the FBG capacity were limited by the sampling hardware. Chen et al. theoretically employed <100 ms real-time interrogation technology in a 2-D sensing network with <275 sensors [12]. In this paper, we propose an effective hardware-based scheme to speed up FBG central wavelength demodulation, especially for over 1000 FBGs. Based on the feature of a weak FBG spectrum, this technique can simultaneously implement central wavelength demodulation for multiple FBGs, and can intrinsically suppress the accumulative noise greatly.

## 2. Principle

**Figure 1:**
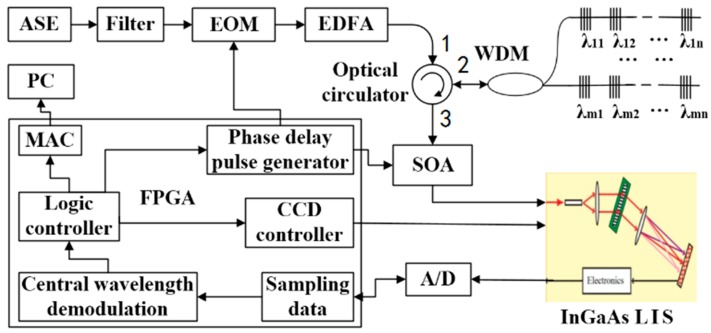
Illustration of interrogation system.

The large-capacity sensing network interrogation system with identical weak FBGs scheme is shown in Figure 1. In the special architecture, the FPGA is the main controller for implementing peak wavelength shift detection. The phase delay pulse generator module modulates the incident light in the sensing network by means of an Electro-optic modulator (EOM), and filters the sensing signals from multiple sensors using a semiconductor optical amplifier (SOA). The InGaAs linear image sensor (LIS) is driven by a CCD controller for photoelectric conversion while the sensing signals are received from SOA. At the same time, the output of InGaAs LIS is sampled by A/D and the central wavelength of the light pulse is calculated in real time. The demodulation results are sent to the upper computer (PC) by the media access controller (MAC). The upper computer (PC) is only responsible for human machine interface (HMI).

The light from amplified spontaneous emission (ASE) is filtered to nanosecond pulses. A pulse generator, realized by FPGA logic, drives the EOM, which functions as a gating device as well as a first-stage optical amplifier. The signal is amplified by the second-stage optical amplifier, an Erbium-doped fiber amplifier, and then fed to port 1 of the optical circulator. Port 2 of the optical circulator directs the signals to the FBG array by wavelength division multiplexing (WDM). The reflected signal from the FBGs returns to the input port of SOA via WDM in port 3 of the circulator. Based on the light reflection time, which is determined based on the spatial location of every FBG along the network, the FPGA generates a time-shifted pulse sequence to activate the SOA to filter and amplify one specific FBG spectrum while absorbing other interface signals, including the reflected spectrum of other FBG sensors. Both the EOM and SOA are controlled by the FPGA logic controller, by applying strict sequential timing constraints, through the fixed phase pulse generator and delay timer, respectively. The InGaAs LIS takes on the role of wavelength demodulator. When the SOA is driven by a time-shifted periodic pulse, the pulse reflected by this FBG will be amplified each time it passes through the SOA. The pulses reflected by other FBGs are blocked. Then, the reflection spectrum of the FBG is measured by the InGaAs LIS. The spectrum of every FBG can be interrogated by changing the delay time. As the SOA is able to work in high-speed switch mode with a minimum switch time of 1 ns, in system initial status, pulse sequences scanning the phase shifter by steps of 1 ns and 0.2 m space resolution, the position of each FBG sensor can be resolved, and the relative phase delay stored. The maximum frequency, which drives the SOA by pulse generator, is defined as the reciprocal of the arriving delay of the FBG at the greatest distance. Generally, the maximum frequency is usually limited by the InGaAs detector demodulation speed if the length of single fiber is less than 5 km.

## 3. Experimental Results

**Figure 2:**
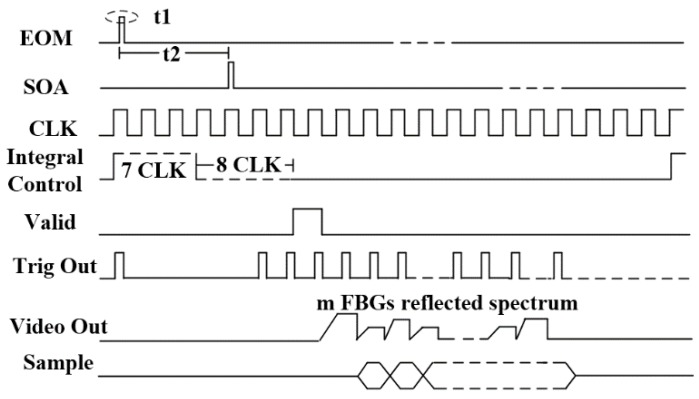
Timing of InGaAs LIS detector and A/D sampling.

The performance of the proposed interrogation system was further verified on a sensor system with three weak FBG arrays. Each array had 143 weak FBGs, positioned at an interval of 2 m, equidistant from one another. The peak reflectivity of the FBGs ranged from −40 dB to −45 dB, and the central wavelengths of the FBGs were nearly the same within each array, but were different among the arrays, at about 1540 nm, 1550 nm, 1560 nm, respectively. The FPGA generated a pulse sequence with width of 20 ns. By changing the delay timer for the SOA, the EOM was driven to generate light pulses that were injected into the FBG array. As a result, the reflected sensing signal from the candidate FBGs with the same optical path difference (OPD) from the circulator were filtered along the sensing fiber, and detected by InGaAs LIS and A/D. The process was controlled by the timing function. Figure 2 shows an example implementation with G11620 InGaAs LIS and AD9826. The pulse width t1 for EOM and SOA refers to the optical path of the FBG distance. The phase delay t2 for SOA was determined by the FBG position along the fiber array. G11620 started to appear when the Integral Control was low, and the integration was completed after eight clocks. After one clock of digital signal processor (DSP) processing, the video data of the electro-optical conversion was sent out to every clock in sequence. AD9826 was configured in advance to transfer all video data to FPGA for processing. Multiple candidate FBGs’ reflected spectra were sampled simultaneously by the InGaAs LIS in the course of one system scanning period.

**Figure 3:**
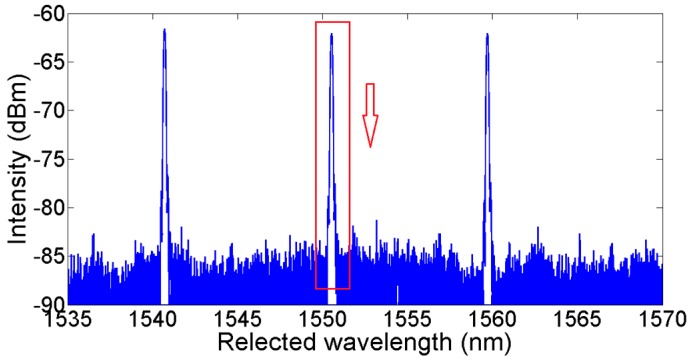

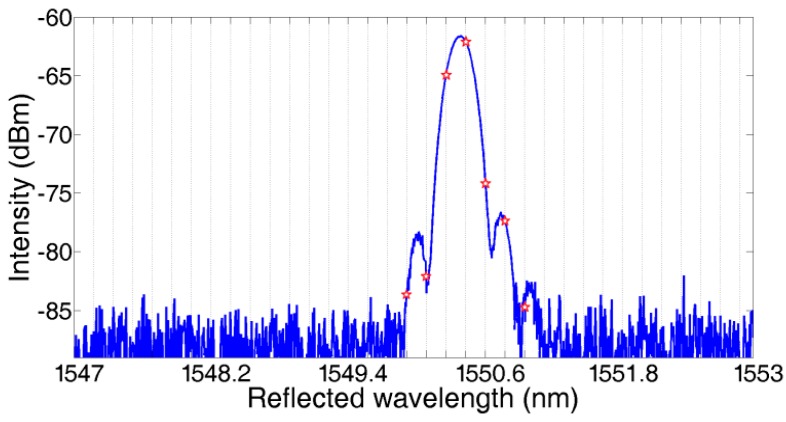
Reflected spectrum of weak FBG.

The reflected spectra of FBG1,1, FBG1,2, and FBG1,3 are shown in Figure 3. Taking FBG1,1 as an example, the seven stars on the waveform are the corresponding valid sample points, which were mapped onto the InGaAs LIS video pixels. As we can see, only three sample points are located on the main lobe, three points on the first-order side lobe, and one sample point on the second-order side lobe. The sample points on the side lobes will not have a favorable result for the central wavelength demodulation. What’s more, with the waveform shifting in applications, these sample points may lead to an incorrect demodulation result.

According to the analysis of the experimental results in [8], the Gaussian fitting algorithm to calculate the central wavelength takes over 0.2 s and the data transmission takes another 0.02 s. Compared to 0.22 s—the average interrogation period—the time for data transmission is negligible in the interrogation scheme proposed in Figure 2, because of all the integrated implementation. Moreover, the data processing time in the electric domain has reduced a lot. The following sections will focus on the implementation of parallel central wavelength calculations to speed up the data processing.

The ${\mathrm{FBG}}_{i,j}$ assumes that the FBG, which is selected randomly, and the reflected spectrum of the ${\mathrm{FBG}}_{i,j}$ can be reconstructed at a different delay time $\tau_{i,j}$ in the time domain window by the sample points set $N_{i,j}=\left[N_{1,i,j},\;N_{2,i,j},N_{3,i,j}\cdots \;N_{s,i,j}\right]$. The Gaussian model is always used to reconstruct the reflected spectrum [13]. The adjusted Gaussian function is shown in Equation (1):

(1) $$f\left(\lambda_{i,j}\right)=A\times \exp\left(-\frac{{\left(\lambda_{i,j}-B\right)}^{2}}{C^{2}}\right)$$

where *A*, *B*, *C* are the adjusted parameters, amplitude, center and deviation, respectively, and $f\left(\lambda_{i,j}\right)$ is the calculated spectrum of $\lambda_{i,j}$. The peak value is equal to *A* when $\lambda_{i,j}=B$.

**Figure 4:**
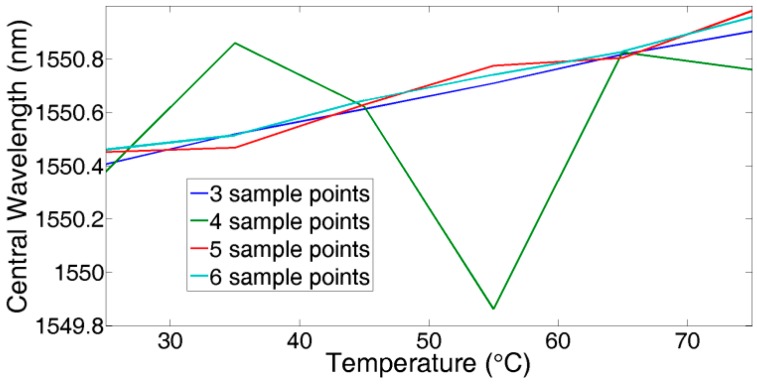
Central wavelength demodulation when temperature changes.

As there are three parameters in Equation (1), theoretically, *A*, *B*, *C* can be derived by three random group values from $N_{i,j}$. However, in fact, from Figure 3, we always get more than three sample points for the reflected spectrum of every FBG. When S > 3, the Gaussian fitting algorithm is expected to obtain the minimum of the sum of squares of deviations (SSE) by the least square method [14]. However, because of the existence of non-main lobe sample points, the Gaussian fitting algorithm does not work well. As Figure 4 shows, the calculated central wavelength shifts with ambient temperature, changing from 25 °C to 75 °C in steps of 10 °C when using different sample points to calculate the central wavelength. From the experimental results, for the reflected spectrum of this FBG, it can be concluded that using three sample points is able to achieve the best linear curve fitting. The results of using over four sample points are less than satisfactory. This agrees with the spectrum analysis conclusion mentioned above.

The equation for implementing central wavelength demodulation in FPGA takes the logarithm of both sides of Equation (2):

(2) $$\mathrm{In}\left(f\left(\lambda_{i,j}\right)\right)=-\frac{{\left(\lambda_{i,j}-B\right)}^{2}}{C^{2}}+\mathrm{In}A$$

The sample point set of the reflected ${\mathrm{FBG}}_{i,j}$ spectrum $N_{i,j}=\left[N_{1,i,j},\;N_{2,i,j},N_{3,i,j}\right]$ can be described based on Equation (3):

(3) $$\lambda_{B,i,j}=B_{i,j}=\frac{1}{2}\times \frac{\left(In(f(\lambda_{2,i,j}))-In(f(\lambda_{3,i,j})))\times (\lambda_{1,i,j}+\lambda_{2,i,j})-(In(f(\lambda_{2,i,j}))-In(f(\lambda_{2,i,j})))\times (\lambda_{3,i,j}+\lambda_{2,i,j}\right)}{\left(In(f(\lambda_{2,i,j}))-(In(f(\lambda_{3,i,j})))+(In(f(\lambda_{2,i,j}))-In(f(\lambda_{2,i,j}))\right)}$$

**Figure 5:**
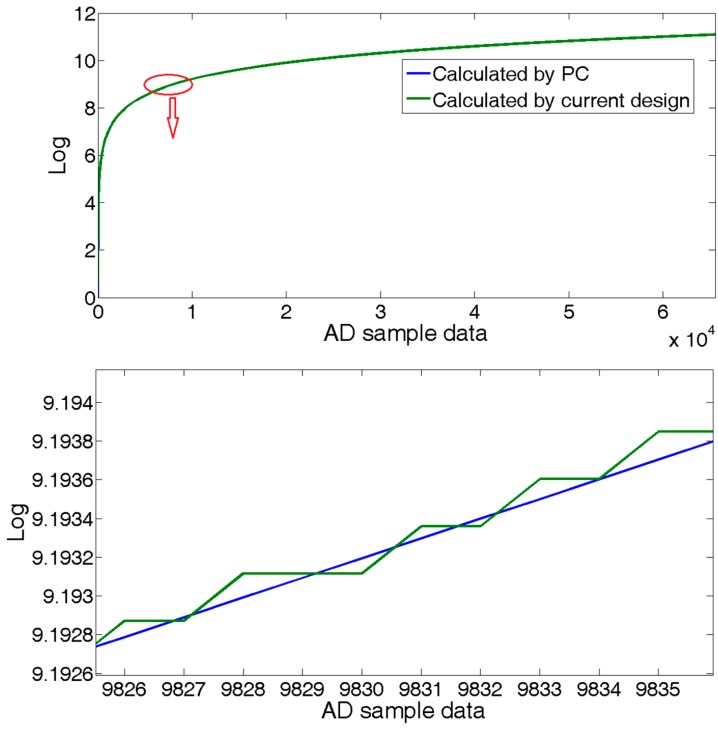
Logarithm calculation result.

To compromise between the occupancy of logic cells and computational accuracy, we use 12-bit for the fractional part in the FPGA logic, which means 0.244 ps error. Figure 5 compares the results of the logarithm calculation between a general 64-bit computer server and FPGA logic with a 16-bit A/D.

**Figure 6:**
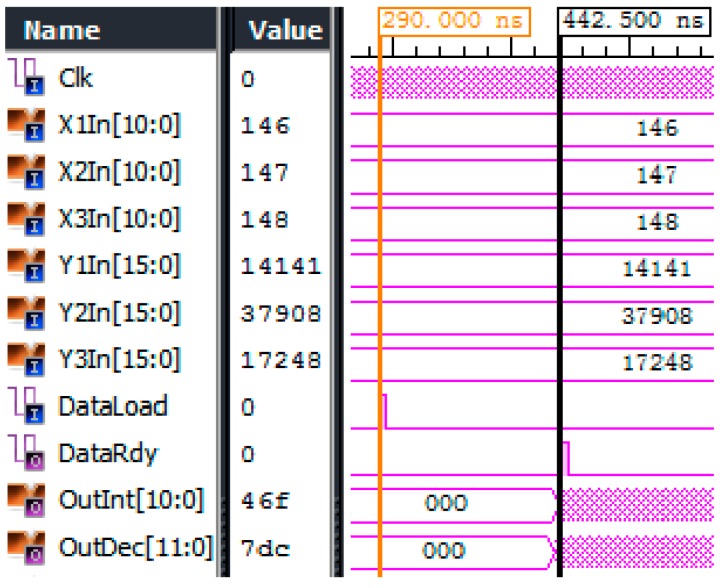
Executive timing of central wavelength demodulation.

The typical execution result of Equation (3) in FPGA is shown in Figure 6. At 200 MHz, the algorithm takes only 152.5 ns. To the authors’ knowledge, this is the fastest FBG central wavelength demodulation method.

In order to avoid multiple pulse interferences, only one laser pulse is allowed in the fiber. Then, the maximum scanning speed of the FBG sensing array is limited by the demodulation time of the sensing signals and the length of the fiber. From Figure 2, InGaAs LIS takes $\frac{8}{f_{CCD}}$ us to integrate the optical density at a working frequency of $f_{CCD}$ MHz. With the pipeline parallel processing technique, the central wavelength demodulation can be completed in the integral timing window by making the Integral Control signal valid just before the outgoing sensing pulse from SOA. The demodulation time of a single FBG is equal to $1/f_{\max}.$ In Equation (1), that is 27.2 us below the maximum 10 MHz work frequency for GS11620. The system scanning period is expressed as Equation (4):

(4) $$t_{i,j}=\{\begin{array}{c} \frac{272}{f_{CCD}},\;L_{i,j}<\frac{4C}{n\times f_{CCD}}\times 10^{-6}m\\ \frac{264}{f_{CCD}}+\;\frac{2{\mathrm{nL}}_{i,j}}{C}\times 10^{6},\;L_{i,j}\geq \frac{4C}{n\times f_{CCD}}\times 10^{-6}m \end{array}$$

where $t_{i,j}$ (unit us) is the arriving delay of the reflected pulse of ${\mathrm{FBG}}_{i,j}$, *c* is the speed of light in a vacuum, *n* is the effective refractive index of the optical fiber, and $L_{i,j}$ is the distance of ${\mathrm{FBG}}_{i,j}$ from the circulator.

**Figure 7:**
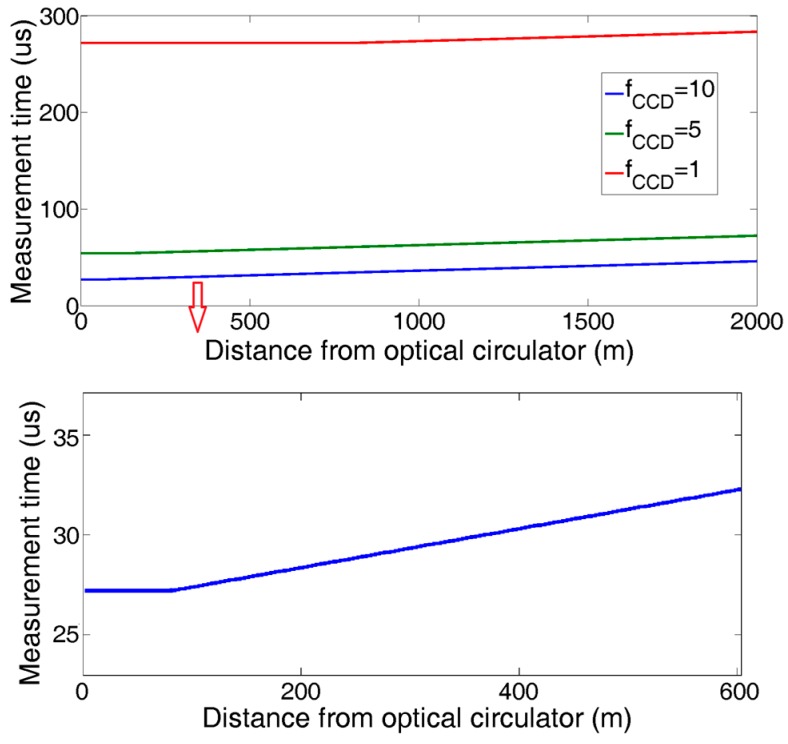
Scanning speed of each sensor on FBG array.

The measured results of all the FBGs along the sensing fiber are shown in Figure 7:

Compared to the previous system, the experimental results verified that the proposed technique reduced the interrogation time by more than 800 times, from an average value of ~0.22 s to a minimum value of 27.2 us. The uncertainty of computer processing made the interrogation time in the previous system unpredictable. However, in the proposed system, the interrogation time of every FBG remains constant, because it is determined by its location along the fiber.

**Figure 8:**
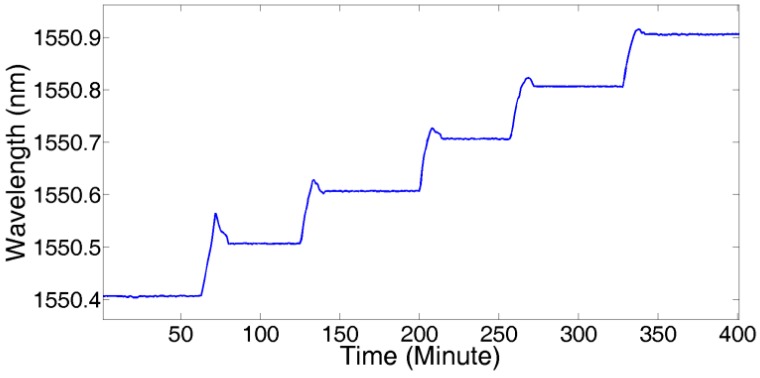
Temperature measurement of ${\mathrm{FBG}}_{1,143}.$

**Figure 9:**
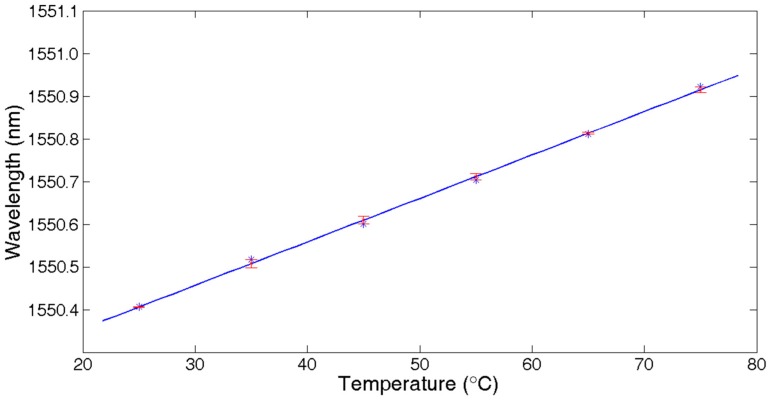
Central wavelength shift vs. temperature change of ${\mathrm{FBG}}_{1,143}.$

To demonstrate the effectiveness of the proposed technique in improving interrogation stability, without loss of generality, we submitted the farthest ${\mathrm{FBG}}_{1,143}$ to a stress test from 25 °C to 75 °C in step of 10 °C in a temperature chamber, as shown in Figure 8:

Figure 9 shows the linear fitting results of temperature with a central wavelength shift of ${\mathrm{FBG}}_{1,143}$. The fitting function was f(x)=0.01083+1550.147, so the temperature sensitivity was about 11 pm/°C.

**Figure 10:**
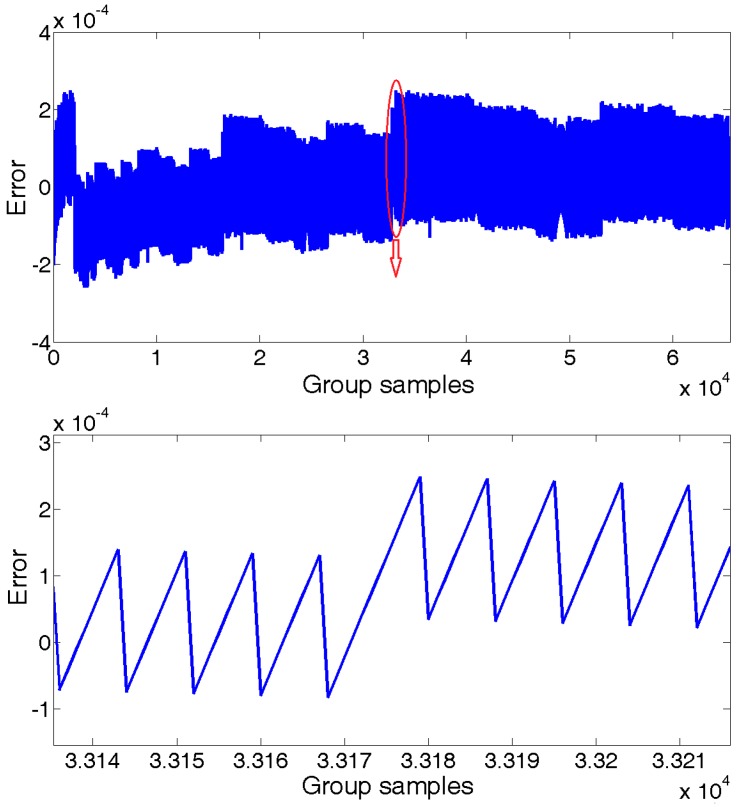
Logarithm calculation error.

Setting the interrogation system provides data at every 100 us, which is an output frequency of 10 kHz. In a period of 7 s, we collected $6.5\times 10^{4}$ group samples of the reflected spectrum data of the sensing FBGs for the central wavelength calculation analysis. Taking the results of the same algorithm executed in a 64 bit server computer for comparison, the proposed technique had about $\pm 2\times 10^{-4}$ calculation error, as shown in Figure 10. Considering the temperature sensitivity of FBGs of about 10 pm/°C, the calculation error would lead to about $\pm 2\times 10^{-5}$ °C measurement error, which can be completely ignored. The proposed technique provides about ±1 pm measurement error without any extra data processing algorithm, such as average.

## 4. Conclusions

In summary, we presented in this paper a novel technique for eliminating the FBG interrogation time in the demodulation scheme of a large-scale FBG sensing array. Using parallel computing and special pipeline control, we developed an ultra-fast central wavelength demodulation method that only uses the valid sample points on the main lobe of the reflected FBG spectrum. The proposed technique was to implement integral controlling logic in FPGA to control InGaAs LIS. This technique is based on the scheme of using EOM and SOA to generate a phase delay to filter the sensing signal from the candidate FBG with the equal optical path to circulator for simultaneous interrogation. The experimental results showed that the proposed technique was able to achieve a minimum interrogation delay time of 27.2 us. Owing to the parallel demodulation of multiple FBG reflection spectra, the system scanning period shortened the corresponding times. The tests also showed that the proposed FPGA-based technique made the FBG wavelength demodulation more stable.

## References

[1] Zhang M.L., Sun Q.Z., Wang Z., Li X.L., Liu H.R., Liu D.M. (2012). A Large Capacity Sensing Network with Identical Weak Fiber Bragg Gratings Multiplexing. Opt. Commun..

[2] Zhu F., Zhang Y.X., Xia L., Wu X.L., Zhang X.P. (2015). Improved Φ-OTDR Sensing System for High-Precision Dynamic Strain Measurement Based on Ultra-Weak Fiber Bragg Grating Array. J. Lightw. Technol..

[3] Fang G.S., Xu T.W., Li F. (2015). Heterodyne interrogation system for TDM interferometric fiber optic sensors array. Opt. Commun..

[4] Liao Y., Austin E., Nash P.J., Kingsley S.A., Richardson D.J. (2013). Highly Scalable Amplified Hybrid TDM/DWDM Array Architecture for Interferometric Fiber-Optic Sensor Systems. J. Lightw. Technol..

[5] Wang S.F., Chen T.H., Tsai L., Huang C.C., Chiang C.C. (2016). A FBG Intensity Modulation System Combined with an Optical Whispering Gallery Mode Edge Filter. Appl. Sci..

[6] Wang Z., Wen H.Q., Luo Z.H., Dai Y.T. (2016). Time Division Multiplexing of 106 Weak Fiber Bragg Gratings Using a Ring Cavity Configuration. Photonic Sens..

[7] Li X.L., Sun Q.Z., Wo J.H., Zhang M.L., Liu D.M. (2012). Hybrid TDM/WDM-Based Fiber-Optic Sensor Network for Perimeter Intrusion Detection. J. Lightw. Technol..

[8] Wang Y.M., Gong J.M., Dong B., Wang D.Y., Shillig T.J., Wang A.B. (2012). A large Serial time-division multiplexed fiber Bragg grating sensor network. J. Lightw. Technol..

[9] Dai Y.B., Li P., Liu Y.J., Asundi A., Leng J.S. (2014). Integrated real-time monitoring system for strain/temperature distribution based on simultaneous wavelength and time division multiplexing technique. Opt. Lasers Eng..

[10] Cui K., Li S.S., Ren Z.J., Zhu R.H. (2017). A Highly compact and efficient interrogation controller based on FPGA for fiber-optic sensor array using interferometric TDM. IEEE Sens. J..

[11] Ai F., Sun Q.Z., Cheng J.W., Luo Y.Y., Yan Z.J., Liu D.M. High accuracy demodulation for twin-grating based sensor network with hybrid TDM/FDM. Proceedings of the 25th International Conference on Optical Fiber Sensors.

[12] Chen X.F., Dong X., Lv H.J., Liu S.C. (2017). Real-time interrogation technology for a large-scale fiber-ring laser sensor array. IEEE Photonics J..

[13] Wang Y.M., Gong J.M., Wang D.Y., Dong B., Bi W.H., Wang A.B. (2011). A quasi-distributed sensing network with time-division-multiplexed fiber Bragg gratings. IEEE Photonics Technol. Lett..

[14] Dai Y.B., Liu Y.J., Leng J.S., Deng G., Asundi A. (2009). A novel time-division multiplexing fiber Bragg grating sensor interrogator for structural health monitoring. Opt. Laser Eng..

